# Bacterial Co-Infections and Antimicrobial Resistance in Patients Hospitalized with Suspected or Confirmed COVID-19 Pneumonia in Kazakhstan

**DOI:** 10.3390/pathogens12030370

**Published:** 2023-02-23

**Authors:** Alyona Lavrinenko, Svetlana Kolesnichenko, Irina Kadyrova, Anar Turmukhambetova, Lyudmila Akhmaltdinova, Dmitriy Klyuyev

**Affiliations:** 1 Research Laboratory, Karaganda Medical University, Karaganda 100008, Kazakhstan; 2 Management Department, Karaganda Medical University, Karaganda 100008, Kazakhstan

**Keywords:** COVID-19 clinical management, living guidance, bacterial co-infections, Kazakhstan

## Abstract

Our study was carried out to characterize respiratory tract microbiota in patients with “COVID-like pneumonia” in Kazakhstan and analyze differences between COVID-19 positive and negative groups. Sputum samples were collected from hospitalized patients, ≥18 years old, in the three cities in Kazakhstan with the highest COVID-19 burden in July 2020. Isolates were identified by MALDI-TOF MS. Susceptibility testing was performed by disk diffusion. We used SPSS 26 and MedCalc 19 for statistical analysis. Among 209 patients with pneumonia, the median age was 62 years and 55% were male. RT-PCR-confirmed SARS-CoV-2 cases were found in 40% of patients, and 46% had a bacterial co-infection. Co-infection was not associated with SARS-CoV-2 RT-PCR test results, but antibiotic use was. The most frequent bacteria were *Klebsiella pneumoniae* (23%), *Escherichia coli* (12%), and *Acinetobacter baumannii* (11%). Notably, 68% of *Klebsiella pneumoniae* had phenotypic evidence of extended-spectrum beta-lactamases in disk diffusion assays, 87% of *Acinetobacter baumannii* exhibited resistance to beta-lactams, and >50% of *E. coli* strains had evidence of ESBL production and 64% were resistant to fluoroquinolones. Patients with a bacterial co-infection had a higher proportion of severe disease than those without a co-infection. The results reinforce the importance of using appropriate targeted antibiotics and effective infection control practices to prevent the spread of resistant nosocomial infections.

## 1. Introduction

The emerging coronavirus infection poses a serious threat to the entire global public health system. The coronavirus infection (COVID-19), which was recorded at the end of 2019, is the third global outbreak in the past two decades [1]; it is also the second pandemic of the twenty-first century according to the World Health Organization (WHO). COVID-19 is caused by the SARS-CoV-2 coronavirus, a new strain from the Coronaviridae family, which was first isolated in Wuhan, China, after a series of outbreaks. This new coronavirus infection has been registered all over the world, except for Antarctica. The new virus causes a spectrum of illnesses, from asymptomatic carriage to severe respiratory distress and death.

From time to time, vaccination throughout the world has become an effective measure to combat coronavirus infection. However, so far there is no complete information on the effectiveness of vaccines against all possible types of coronavirus infection [2].

Current treatments for coronavirus infection target cytokine-releasing drugs [3]. At the same time, other methods of treatment are used, such as antivirals, antimalarials, antibacterial drugs, immunomodulators, angiotensin II receptor blockers, bradykinin B2 receptor antagonists, corticosteroids, antiprotozoals, anticoagulants, and others. The management of patients with coronavirus infection remains largely supportive and is under constant research and adaptation [4].

Antimicrobial resistance (AMR) is a growing global health threat exacerbated by the COVID-19 pandemic. The pandemic has caused surges in hospitalizations coupled with intense usage of invasive devices and antimicrobial treatment creating settings prime for the spread of bacterial resistance. Bacterial respiratory co-infection in patients hospitalized with coronavirus infection is associated with poor clinical outcomes, especially when bacteria are resistant to first-line antimicrobial treatment [5,6,7,8,9]. Antibiotics are frequently administered to patients with suspected or confirmed coronavirus infection even though they are ineffective for treating viral infections and only a small percentage of patients with bacterial infections need antibiotic therapy [10,11]. Moreover, patients with coronavirus infection who receive antibiotics do not have improved outcomes, and empiric antibiotic therapy can result in the growth of resistant strains and worsen the course of viral pneumonia [12,13].

There has been worldwide concern regarding the accelerated growth and spread of resistant microorganisms during the COVID-19 pandemic [11] that can weaken global efforts to fight drug-resistant infections, especially in settings lacking laboratory diagnostics where an inappropriate antimicrobial prescription is commonplace, such as Kazakhstan [14,15]. Although antimicrobial stewardship has increased in Kazakhstan, antibacterial drugs are still commonly dispensed inappropriately throughout the country and have been used empirically for the treatment of coronavirus-infection-associated pneumonia [16]. However, diagnosis and treatment protocols for coronavirus infection are continually being revised as more information becomes available [17].

Data on secondary bacterial respiratory infections and levels of antimicrobial resistance among hospitalized cases are needed to inform these protocols. For this purpose, our study was carried out to characterize respiratory tract microbiota in patients with “COVID-like pneumonia” in Kazakhstan and find differences between COVID-19 positive and negative groups.

## 2. Materials and Methods

### 2.1. Study Participants

We conducted a prospective, microbiological, multicenter study, at the Medical University of Karaganda Research Laboratory in Kazakhstan in July 2020. The study included hospitalized patients, ≥ 18 years old, with clinical characteristics of coronavirus-infection-associated pneumonia categorized by severity of disease [18]:

- Mild Illness: Individuals who have any of the various signs and symptoms of coronavirus infection (e.g., fever, cough, sore throat, malaise, headache, muscle pain, nausea, vomiting, diarrhea, loss of taste and smell) but who do not have shortness of breath, dyspnea, or abnormal chest imaging.
- Moderate Illness: Individuals who show evidence of lower respiratory disease during clinical assessment or imaging and who have an oxygen saturation (SpO_2_) ≥ 94% on room air at sea level.
- Severe Illness: Individuals who have SpO_2_ < 94% on room air at sea level, a ratio of arterial partial pressure of oxygen to fraction of inspired oxygen (PaO_2_/FiO_2_) < 300 mm Hg, respiratory frequency > 30 breaths / min, or lung infiltrates > 50%.
- Critical Illness: Individuals who have respiratory failure, septic shock, and/or multiple organ dysfunction.

Participants were selected from three dispensary hospitals in the three cities in Kazakhstan that had the highest coronavirus infection case load (Almaty, Karaganda, and Atyrau) during the July 2020 peak. All patients hospitalized with bilateral community-acquired pneumonia with a reverse transcription polymerase chain reaction (RT-PCR) laboratory-confirmed SARS-CoV-2 test (group 1) and negative SARS-CoV-2 RT-PCR test (group 2) were included in the study. RT-PCR testing was performed at each city’s National Centre of Expertise of the Committee of Sanitary and Epidemiological Control of the Ministry of Health of the Republic of Kazakhstan (NCE). Clinical data collected included: sex, age, date of symptom onset, date of diagnosis of bilateral pneumonia, hospitalization, and SARS-CoV-2 RT-PCR test result, computed tomography result showing percentage lung damage, antibiotic drug therapy, and category of the severity of disease as defined by the World Health Organization [19].

### 2.2. Laboratory Testing

Sputum samples were collected from patients within 48 h of admission to the dispensary hospital to exclude patients with nosocomial pneumonia. Specimens were transported to the Research Laboratory at the Medical University of Karaganda within 4 h of collection. Microbiological processing was carried out in accordance with standard microbiological methods [20]. Prior to identification, isolates were stored at −70 °C in trypticase soy broth supplemented with 30% glycerol.

Isolates were identified to species level by matrix-assisted laser desorption ionization–time-of-flight mass spectrometry (MALDI-TOF MS) using the Microflex LT system and the MALDI Biotyper Compass 4.1.80 software (Bruker Daltonics, Bremen, Germany). The value of “score” ≥ 2.0 was accepted as the measure for identification.

Susceptibility testing was performed by disk diffusion method on Mueller–Hinton agar (Franklin Lakes, NJ, USA) with the addition of 5% sheep blood, depending on the pathogen, in accordance to the Clinical and Laboratory Standards Institute (CLSI M100-24). Results were entered into WHONET 5.6 and interpreted in accordance with the CLSI criteria (2018) [21].

Phenotypic detection of methicillin-resistant *Staphylococcus aureus* (MRSA) was conducted by cefoxitin disk (30 μg). MRSA positive strains had zone of inhibition ≤ 23 mm [21].

ESBL producers were determined by using ceftazidime (30 μg), amoxicillin/clavulanic acid (20/10 μg), and cefotaxime (30 μg) disks [21].

To control for quality of sensitivity testing, *Escherichia coli* ATCC^®^25922, *Escherichia coli* ATCC^®^35218, *Staphylococcus aureus* ATCC^®^29213, *Enterococcus faecalis* ATCC^®^29212, and *Pseudomonas aeruginosa* ATCC^®^27853 strains were used.

### 2.3. Data Analysis

Statistical processing of measurements was carried out using the SPSS 26 (SPSS Inc., Chicago, IL, USA) and MedCalc 19 software. Fisher’s χ^2^, Student’s t-test were used to detect differences between patients with or without coronavirus infection.

The ethics committee of Karaganda Medical University #45 from 6 April 2020 approved the analysis.

## 3. Results

Among 209 patients hospitalized with suspected or confirmed COVID-19 pneumonia, 64 (31%) were from Almaty, 50 (24%) from Atyrau, and 95 (45%) from Karaganda (Table 1). The mean age was 60 years (±14), 55% were male, 56% had positive SARS-CoV-2 RT-PCR test results, and 90% were receiving antibiotic treatment. The mean volume of lung damage in patients was 44% (±21). Sputum cultures showed no growth in 30% of patients and normal microbiota in 24% of patients, mainly *Streptococcus* spp. and coagulase-negative Staphylococci. Of the 97 patients (46%) with bacterial co-infections, 11 had more than one pathogen detected in their sputum.

The proportion of patients with positive SARS-CoV-2 RT-PCR did not differ by city, but more women than men had positive SARS-CoV-2 RT-PCR (52% vs. 38%, *p* = 0.03). Among participants with negative SARS-CoV-2 RT-PCR, the volume of lung damage was greater, 54% vs. 43%, than those with positive results, though this difference was not significant (*p* = 0.13). A greater proportion of patients with confirmed coronavirus infection were on antibiotics than those not confirmed (97% vs. 83%, *p* < 0.01). Antibiotic use was associated with the detection of *P. aeruginosa* in two out of three samples (66%, *p* = 0.02), *S. aureus* in 7 out of 10 samples (70%, *p* < 0.01), and *Candida* spp. in 67 out of 124 samples (54%, *p* = 0.03).

The differences in identified isolates were not affected by COVID status. Enterobacterales accounted for 44% of all isolated strains, of which 15% were *K. pneumoniae* and 9% were *E. coli*. Of the non-fermenting microorganisms, *A. baumannii* was isolated in 11%, and *P. aeruginosa* and *S. maltophilia* in 2%. Of the Gram-positive microorganisms, *S. aureus* was isolated in 10% of cases, as well as the classic causative agent for pneumonia, *S. pneumoniae*, in 1%. In 15% of cases, fungi of the genus *Candida* were isolated. There were also no differences in microorganisms cultured in each city, except for *Streptococcus pneumoniae* (*n* = 3), which was isolated only in samples obtained from Karaganda city.

All patients with *A. baumannii*, *S. maltophilia*, and *S. pneumoniae*, and 94% of patients with *K. pneumoniae* had severe or critical pneumonia. This difference was statically significant for *A. baumannii* and *K. pneumoniae*.

These microorganisms alone, without concomitant viral infection, pose a danger to an impatient. *A. baumannii, S. maltophilia,* and *K. pneumoniae* are common nosocomial pathogens worldwide, while in patients with normal microbiota [22], only 74% had severe pneumonia (Table 2).

**Figure 1:**
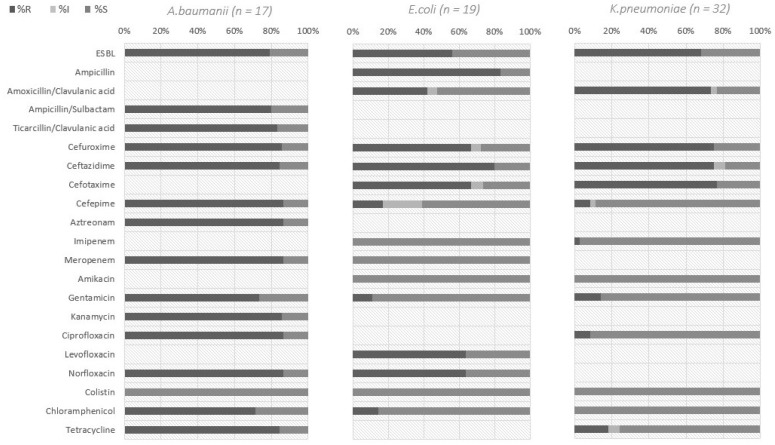
Susceptibility profiles of isolates from sputum samples of patients hospitalized with suspected or confirmed COVID-19 pneumonia in 3 hospitals in Kazakhstan, June 2020. %R—percentage of resistant strains; %I—percentage of intermediate strains; %S—percentage of susceptible strains.

Among isolated pathogens, elevated levels of antibiotic resistance were noted (Figure 1). Among the *K. pneumoniae* isolates (*n* = 32), 68% had phenotypic evidence of extended-spectrum beta-lactamases (ESBL) production, 14% were resistant to aminoglycoside group drugs (gentamicin), 9% were resistant to fluoroquinolones, and 3% were resistant to imipenem. No resistance to chloramphenicol and polymyxins was detected. More than 50% of *E. coli* strains had the phenotypic features of ESBL production, and 64% had resistance to fluoroquinolones (levofloxacin and norfloxacin). All *E. coli* strains were susceptible to the carbapenems group. In total, 11% of *E. coli* strains were resistant to gentamicin, and 14% to chloramphenicol. Among *A. baumannii* isolates (*n* = 17), 87% had resistance to beta-lactams, cefepime, and meropenem, and 87% were resistant to ciprofloxacin, norfloxacin, and aztreonam. In total, 73% of *A. baumannii* samples were resistant to gentamicin, and 85% to tetracycline. *A. baumannii* strains were highly sensitive to polymyxin/colistin.

*P. aeruginosa* strains (*n* = 3) were characterized by high sensitivity to antimicrobial drugs, except for second and third generation cephalosporins. *S. maltophilia* isolates (*n* = 3) were resistant to most currently used antibiotics, including β-lactams, carbapenems, cephalosporines, chloramphenicol, and tetracyclines. *S. pneumoniae* isolates (*n* = 2) had high resistance to fluoroquinolones. Notably, one strain was resistant to 14-,15-member ring macrolides, and lincosamides. Of 10 *S. aureus* strains, 20% were identified as methicillin-resistant *Staphylococcus aureus* (MRSA), and were also resistant to tetracycline, azithromycin, gentamicin, and ciprofloxacin. No resistance to vancomycin, rifampicin, fusidic acid, and linezolid was observed.

## 4. Discussion

We analyzed bacterial co-infections in patients hospitalized with suspected or confirmed COVID-19 pneumonia in July 2020 in Kazakhstan. To our knowledge, this is the first study to identify respiratory bacterial co-infection among patients with confirmed or probable COVID-19 pneumonia in Kazakhstan. COVID-19 secondary infections have been described by our foreign colleagues in previous studies based on the example of hospitalized patients with coronavirus infection [6]. The incidence of secondary infection in these studies varied depending on the definition criteria and the heterogeneity of the inclusion of patients in the sample, as well as the diagnostic methods used in the study [10,23].

We observed a high proportion of patients in our study that had bacterial co-infections (71%); a proportion substantially higher than that of studies from nearby countries (5% and 10% in two studies in China) [5,6,24,25].

At the moment, the literature describes quite a large number of data on the bacterial composition of the secondary infection with coronavirus infection. Some sources describe the occurrence of a secondary infection caused by Gram-negative microorganisms (Enterobacterales, *A. baumannii*, *P. aeruginosa)* [10,26]. Other sources describe the role of *Mycoplasma pneumoniae*, *P. aeruginosa*, *H. influenzae*, and *Klebsiella* spp. [6] as causative agents of secondary infection in COVID-19 pneumonia.

In our study, isolated microorganisms belong to the ESKAPE group of pathogens (*Enterococcus faecium*, *Staphylococcus aureus*, *Klebsiella pneumoniae*, *Acinetobacter baumannii, Pseudomonas aeruginosa*, and *Enterobacter* spp.), six highly virulent and antibiotic-resistant bacterial pathogens that represent a significant global threat to human health [27]. Patients with these co-infections in our study had the highest risk of severe or critical disease. The results are consistent with the literature showing that mortality and morbidity are higher among patients with coronavirus infection who have concomitant bacterial infections than among those who do not [28,29].

We also found high levels of resistance among pathogens detected in our study. Morbidity and mortality associated with COVID-19-associated pneumonia is further increased when drug-resistant bacteria are present [24,26,29,30].

Inappropriate widespread empirical use of broad-spectrum antibiotics for treating coronavirus infection in patients with suspected or confirmed disease may have contributed to the observed resistance patterns [26].

Unfortunately, the observed high levels of ESBL-producing strains of *K. pneumoniae* and *E. coli* are becoming common in our hospitals. ESBL-producing strains of Enterobacterales often possess genes conferring resistance to other classes of antimicrobials, including fluoroquinolones and aminoglycosides [31]. A similar situation is observed in the present study, where ESBL-producing strains of Enterobacterales were characterized by resistance to the groups of antibiotics listed above. Surveillance for antimicrobial resistance should be implemented to stop the spread of Enterobacterales, which produce extended-spectrum beta-lactamases. However, beta-lactam antimicrobials are the most commonly used antibiotics in hospitals [32]. Accordingly, their inefficiency is a big problem for our healthcare system, requiring additional patient stays in the hospital, as well as additional funding [33].

Resistance patterns in *A. baumannii* isolated were especially concerning. *A. baumannii* infections are a common healthcare-associated infection with very low prognosis for critically ill patients [34]. The strains isolated had resistance to carbapenems, which are a serious threat to the healthcare system. Few treatment options are available for treating carbapenem-resistant *A. baumannii*. Colistin is a last-resort antimicrobial drug for treating carbapenem resistance, and fortunately, the strains isolated in our study showed no resistance to colistin. A lower than expected proportion of isolates were MRSA (20%). *S. aureus* infections are common in hospitals in the region and are associated with high mortality rates among patients in intensive care units with lower respiratory tract infections [35,36,37].

The role of secondary infection in the development of complications of COVID-19 pneumonia is still a matter of debate. Given our results, namely the high level of bacterial flora in patients with COVID-19 pneumonia, further similar studies should be carried out to determine the levels of bacterial co-infection in viral pneumonia.

Our study shows that isolated secondary bacterial pathogens are quite dangerous for patients because they are classic nosocomial pathogens with a high level of resistance to antibacterial drugs. The coronavirus infection pandemic reaffirms the importance of infection control and the prudent use of antimicrobials in hospitals to all of us.

In this regard, it is necessary to implement programs for the rational use of antimicrobials and the improvement in infection control measures in hospitals.

Our study is subject to at least two limitations. First, we were unable to genotype isolates to detect specific resistance genes. Without genotyping, we are unable to detect the presence of specific resistance genes that can be horizontally transferred across bacteria. Resistance to carbapenems, as detected with *A. baumannii* in our study, is often associated with specific genes. Second, the number of samples was not as big as expected, so it was not enough to compare regional differences.

Our results highlight the need for close monitoring of nosocomial infections and secondary infections to reduce the risk of death or severe disease in patients with COVID-19-associated pneumonia [38]. In the course of our study, highly resistant strains belonging to the ESKAPE group were found, which limits the choice of available antibacterial drugs.

This fact indicates the need to adhere to rational antibacterial therapy among coronavirus infection patients, as well as more thorough epidemiological surveillance and monitoring of antibiotic resistance in the healthcare sector of the Republic of Kazakhstan.

## Figures and Tables

**Table 1:** Demographic and clinical characteristics of patients hospitalized with RT-PCR positive and negative COVID-19 pneumonia (*n* = 209) in Almaty, Atyrau, and Karaganda, Kazakhstan, July 2020.

	Total	SARS-CoV-2 RT-PCR Result		*p*-Value
		(+)	(−)	
	*n* (%) or (SD)	*n* (%) or (SD)	*n* (%) (SD)	
Total	209	116 (56)	93 (44)	0.07
City				0.7
Almaty	64 (31)	33 (28)	31 (33)	0.5
Atyrau	50 (24)	25 (22)	25 (27)	0.4
Karaganda	95 (45)	58 (50)	37 (40)	0.1
Male	114 (55)	56 (48)	58 (62)	0.5
Female	95 (45)	60 (52)	35 (38)	0.03 *
Age, median [range] years	60 (14)	61 (13)	60 (14)	0.4
% lung damage, mean [SD]	44% (21)	43% (21)	54% (26)	0.1
On antibiotics	189 (90)	112 (97)	77 (83)	<0.01 *
Sputum sample results				
No growth	62 (30)	−	−	−
Normal microbiota	50 (24)	28 (24)	22 (24)	0.7
Pathogenic bacteria detected	97 (71)	50 (43)	47 (51)	
Species of isolates (*n* = 106)				
All Enterobacterales	70 (44)	34 (29)	36 (39)	0.9
*Klebsiella pneumoniae*	32 (15)	2 (2)	1 (1)	0.9
*Acinetobacter baumannii*	17 (11)	7 (6)	10 (11)	0.4
*Escherichia coli*	19 (9)	12 (10)	7 (8)	0.6
*Pseudomonas aeruginosa*	3 (2)	2 (2)	1 (1)	0.9
*Stenotrophomonas maltophilia*	3 (2)	2 (2)	1 (1)	0.6
*Staphylococcus aureus*	10 (6)	6 (5)	4 (4)	0.9
*Streptococcus pneumoniae*	3 (1)	2 (2)	1 (1)	0.9
*Candida* spp.	124 (15)	73 (63)	51 (55)	0.2

*—Statistical significance if *p* < 0.05.

**Table 2:** Pneumonia severity among patients hospitalized with suspected or confirmed COVID-19 pneumonia by microorganism isolated in sputum samples, Kazakhstan, June 2020.

Species Cultured	*N*	Disease Severity Classification				
		Mild or Moderate		Severe or Critical		
		*n*	%	*n*	%	*p*-Value
**Normal Microbiota [22]**	50	13	26%	37	74%	0.4
*A. baumannii*	17	0	0%	17	100%	<0.01 *
*P. aeruginosa*	3	2	66%	1	33%	0.5
*S. maltophilia*	3	0	0%	3	100%	0.3 ^b^
*K. pneumoniae*	32	2	6%	30	94%	<0.01 *
*E. coli*	19	5	26%	14	74%	0.9
**Other Enterobacterales** ^a^	19	3	16%	16	84%	0.3
*S. aureus*	10	4	40%	6	60%	0.9
*S. pneumoniae*	3	0	0%	3	100%	0.3^b^
*Candida* spp.	124	39	23%	95	77%	0.9

^a^—Other Enterobacterales: *Klebsiella oxytoca*, *Klebsiella ornithinolytica*, *Enterobacter cloacae, Enterobacter asburiae*, *Proteus mirabilis*; ^b^—adjusted to zero and 100% effect; *—Statistical significance if *p* < 0.05.

## Data Availability

The datasets generated and analyzed during the current study are available from the corresponding author on reasonable request.
